# Toxicity of Fatty Acid Autoxidation Products: Highest Anti-Microbial Toxicity in the Initial Oxidative Phase

**DOI:** 10.3390/molecules20010035

**Published:** 2014-12-23

**Authors:** Jorma Matikainen, Markku Lehtinen, Eila Pelttari, Hannu Elo

**Affiliations:** 1 Laboratory of Organic Chemistry, Department of Chemistry, University of Helsinki, P.O. Box 55 (A. I. Virtasen aukio 1), FI-00014 Helsinki, Finland; E-Mail: jorma.matikainen@helsinki.fi; 2 Division of Pharmaceutical Biosciences, Faculty of Pharmacy, University of Helsinki, P.O. Box 56 (Biocenter 1, Viikinkaari 9), FI-00014 Helsinki, Finland; E-Mails: markku.t.lehtinen@helsinki.fi (M.L.); eila.pelttari@helsinki.fi (E.P.)

**Keywords:** polyunsaturated fatty acids, fatty acid esters, autoxidation, weighing method, antibacterial activity, antifungal activity, toxicity, α-linolenic acid methyl ester

## Abstract

The autoxidation-degradation processes of polyunsaturated fatty acids give rise to toxic products, and the relative toxicity at different stages of the process is of great interest. We report here that when methyl α-linolenate is exposed to sunlight and air, its antimicrobial activity against yeasts and bacteria (as measured by agar diffusion) reaches its maximum during the early oxidative phase when addition of oxygen occurs and the mass increases drastically. Before exposure, the activity is minimal or zero, but it increases rapidly during the first days of the test, simultaneously with the increase of the mass of the material, and begins to decrease while the mass is still increasing and before the mass begins to decrease due to degradation and formation of volatile compounds. Thus, the products formed during the degradation phase of the process are far less toxic to the test organisms than the compounds formed at the early stages when addition of oxygen occurs with maximal rate.

## 1. Introduction

The autoxidation and the accompanying degradation of polyunsaturated fatty acids and their esters gives rise to toxic products [1,2,3], and it would be of interest to know exactly the relative toxicity (or other biological effects) of the material in question at different stages of the autoxidation-degradation process.

The antifungal and antibacterial properties of fatty acids are well known [4]. Free fatty acids are known to be able to kill or inhibit the growth of bacteria, and their antibacterial properties are used by many organisms to defend against bacteria [5]. The mode of action of free fatty acids is not well understood, but their action may result e.g., from autoxidation products. Thus, direct studies on the effects of autoxidation on the antimicrobial properties of fatty acids and their derivatives and, especially, on the toxicity of the products formed at different stages of the autoxidation process are warranted.

A well-known, simple and practical weighing method for following the autoxidation of polyunsaturated lipids, based on the weight changes that occur while oxygen binds to the materials and, at later phases of the process, degradation and formation of volatile substances takes place, has been previously applied to a study of the autoxidation of methyl α-linolenate (ML) and linoleate [6]. This method does not require complicated chemical analyses to be performed. We have now applied that method to the systematic study of the antimicrobial effects of ML against bacteria and yeasts during the course of the autoxidation and degradation process that occurs when the ester is exposed to sunlight and air.

## 2. Results and Discussion

ML was exposed to light and its degree of oxidation was monitored by repeated weighing during a period of several weeks (see the Experimental Section for details). At times, samples were taken for antimicrobial activity measurements against four different microbial strains (the yeasts *Candida albicans* and S*accharomyces cerevisiae*, the Gram-negative bacterium *Escherichia coli* and the Gram-positive bacterium *S. aureus*).

**Figure 1:**
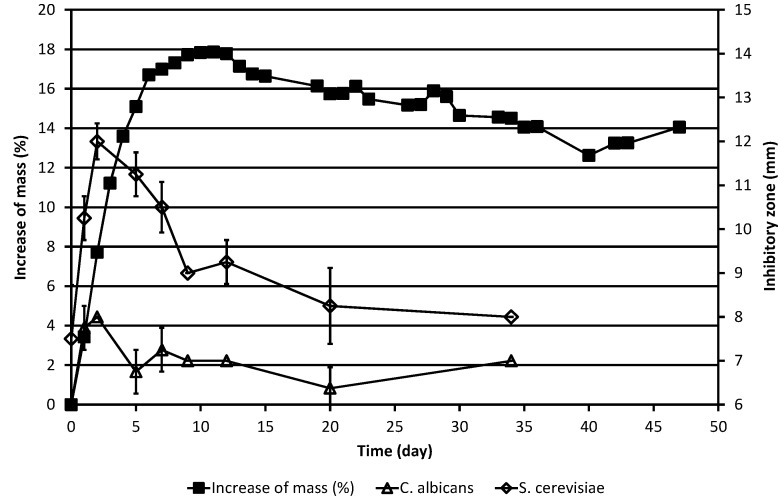
Increase of the corrected mass of the ML sample during the autoxidation process, as compared to the original mass, and the results of simultaneous determinations of inhibitory zones against the yeasts *C. albicans* and *S. cerevisiae*. The corrected mass of the ML was calculated using Equation (1). In cases where no error bar is shown for the inhibitory zone value, the standard deviation was zero.

**Figure 2:**
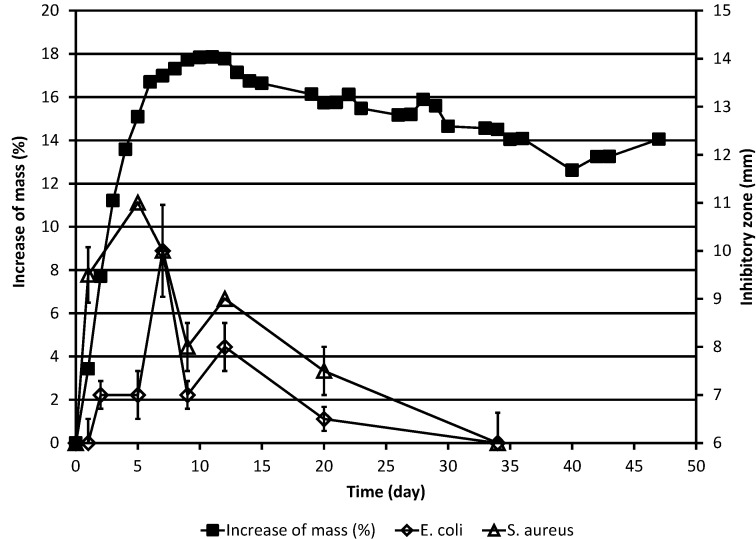
Increase of the corrected mass of the ML sample during the autoxidation process, as compared to the original mass, and the results of simultaneous determinations of inhibitory zones against the bacteria *E. coli* and *S. aureus*. The corrected mass of the ML was calculated using Equation (1). In cases where no error bar is shown for the inhibitory zone value, the standard deviation was zero.

The results of the weighing experiments and those of the simultaneous determinations of the inhibitory zones against the yeasts and against the bacteria, respectively, are shown in Table 1 and in Figure 1 and Figure 2. Because samples were taken from the Petri dish, the weighing results must be corrected for the loss of material due to sampling. This was done using Equation (1):

(1) $$\;m_{n}^{cor\;}=\;m_{n}^{0\;}\times \;\overset{n-1}{\underset{i=0}{\prod }}\frac{m_{i}^{0}}{m_{i}^{r}}$$

where $m_{n}^{cor\;}$ indicates the corrected mass of the ML on day *n* before sampling for microbiological tests, $m_{n\;}^{0}$ indicates the uncorrected mass (*i.e.*, measured mass) of the ML on day *n* before sampling, $m_{i}^{0}$ indicates the uncorrected mass on day *i* before sampling, and $m_{i}^{r}$ indicates the measured mass on day *i* after sampling.

**Table 1:** Inhibitory zones as determined during the autoxidation process of ML ^a^.

Day	Inhibitory Zone Diameter (mm) ± S.D ^b^			
	*C. albicans*	*S. cerevisiae*	*E. coli*	*S. aureus*
0	6 ± 0	7.5 ± 1	6 ± 0.2	6 ± 0
1	8 ± 0.4	10 ± 0.4	6 ± 0.4	9.5 ± 0.5
2	8 ± 0	12 ± 0.4	7 ± 0.3	ND ^c^
5	7 ± 0.4	11 ± 0.4	7 ± 0.4	11 ± 0
7	7 ± 0.4	10.5 ± 0.5	10 ± 0.8	10 ± 0.4
9	7 ± 0	9 ± 0	7 ± 0.3	8 ± 0
12	7 ± 0	9 ± 0.4	8 ± 0.4	9 ± 0.4
20	6.5 ± 0.4	8.5 ± 0.8	6.5 ± 0.2	7.5 ± 0.5
34	7 ± 0	8 ± 0	6 ± 0	6 ± 0

Notes: ^a^ A solution of the ML autoxidation reaction sample was dissolved in dimethyl sulfoxide (DMSO) so that the concentration was 40 mg/mL DMSO. Ten µL of this solution was pipetted on the filter paper disc (diameter of disc 6 mm). Four determinations were carried out in each case. The antifungal drug amphotericin B (8 mg/mL in water) gave inhibitory zone diameters of 21.0 ± 1.2 mm and of 12.9 ± 1.4 mm in the case of *C. albicans* and *S. cerevisiae*, respectively, and the antibiotic cefuroxime (5 mg/mL in water) those of 30.8 ± 0.9 mm (*S. aureus*) and 33.3 ± 1.3 mm (*E. coli*), with *n* = 40 in each case.; ^b^ All results are arithmetic means of four individual measured values and have been rounded to the nearest integer or half-integer; ^c^ ND = could not be determined (technical problem).

As shown in Figure 1 and Figure 2, the (corrected) mass of the ML behaved essentially as previously described [6], increasing first rapidly during the first five days and then more slowly till day 11, after which it began to slowly decrease.

As is evident from Table 1 and Figure 1, ML already had slight antifungal activity against baker’s yeast, *S. cerevisiae*, in the agar diffusion test system at the beginning of the experiment, the inhibitory zone being *ca.* 7.5 mm, but displayed no activity against the other yeast studied, the pathogen *C. albicans*. In the case of *S. cerevisiae*, the antifungal activity increased drastically during the next two days, with the inhibitory zone reaching 12 mm on day 2. Then, the activity began to decline, but there was detectable activity even on day 34. In the case of *C. albicans*, only quite modest activity was observed on days 2 and 3. Thereafter, still lower activity was displayed till the end of the experiment on day 34.

The effects of the test substance against the bacteria *E. coli* and *S. aureus* as a function of time are shown in Table 1 and Figure 2. In the case of both bacteria, the overall results are qualitatively largely similar to those obtained for the yeasts. For both bacteria, the antimicrobial activity was lower than in the case of *S. cerevisiae* but higher than in the case of *C. albicans* and maximal activity appears to be reached somewhat later than in the case of the yeasts. In interpreting inhibitory zone results it should, however, be borne in mind that small (1–2 mm) differences between diameters of inhibitory zones need not necessarily be significant since natural variation may always affect the results of antimicrobial activity studies.

The mechanisms responsible for the slight initial activity of ML against *S. cerevisiae* remain obscure at present, just as do the reasons behind the differential effects on the different microbial strains. Possibly, the differences might be related e.g., to metabolic differences or to differential uptake of ML and its oxidation products into the different microbes.

In MIC tests, no antimicrobial activity could be observed against *C. albicans* or *S. cerevisiae* in two-day cultures (MIC always >1000 µg/mL). In one-day cultures, MIC was ≥1000 µg/mL for all samples taken on day 2 or later as well as for samples of deep-frozen ML. For samples taken on day 1, the MIC value for *C. albicans* was ≥250 µg/mL and that for *S. cerevisiae* ≥125 µg/mL (results for higher concentrations remained inconclusive because of formation of a lipid film on top of the wells).

Likewise, in one-day MIC tests, neither the deep-frozen ML sample (that corresponds to the day 0 sample) nor any one of the samples taken on day 2 or later displayed any antibacterial activity against *E. coli* (MIC > 1000 µg/mL). The sample taken on day 1 did not display any activity at concentrations ≤125 µg/mL (results on higher concentrations remaining inconclusive). In the case of *S. aureus*, the one-day MIC value was ≥1000 µg/mL for the initial sample as well as for samples taken on days 5, 9, 12 and 20. For the samples taken on days 2 and 34, the MIC value was *ca.* 250–500 µg/mL, and for that taken on day 7 it was 500–1000 µg/mL.

The results obtained indicate that biological effects of the materials formed at different stages of the autoxidation-degradation process can be easily studied by using the weighing method for following the process. The agar diffusion study results indicate that the antimicrobial activity of the material formed in the autoxidation-degradation process of ML increases rapidly and reaches its maximum during the early phase of the process when the mass of the substance is also increasing rapidly due to the addition of oxygen.

Further, the results indicate that in MIC tests, the samples generally displayed no or, at best, very low activity, in contrast to the agar diffusion tests where distinct and time-dependent activity was observed. One reason for the lack of antimicrobial activity in the MIC tests may be constituted by the fact that in these tests at least some and, possibly, even a major fraction of the test substance floated on the surface of the aqueous medium in the well, being thus unavailable to the microbial cells. This is in contrast to the case of the agar diffusion tests, in which the relative amount of DMSO in the critical area at and around the paper disc is at least in the beginning of the experiment far higher than in the bulk of the aqueous medium in the MIC test wells, facilitating higher solubility of the lipids. Thus, in studying antimicrobial effects of lipids, the agar diffusion method is clearly superior to the MIC method and liquid culture methods in general.

## 3. Experimental Section

### 3.1. Methyl Linolenate

ML was prepared by acid-catalyzed transesterification with methanol from linseed oil because the fatty acid residues of this oil are known to consist largely (*ca.* 60%) of α-linolenic acid. Trace amounts of concentrated sulfuric acid were employed as the catalyst. The ester was purified essentially as previously described [6,7] using argentation chromatography followed by normal flash silica chromatography. The purity of the product was verified with the aid of ^1^H-NMR and thin-layer argentation chromatography using a TLC plate (TLC Silica gel 60 F_254_, product number 1.05554.0001, Merck, Darmstadt, Germany) impregnated with AgNO_3_ by first dipping in a solution containing 10% AgNO_3_ in acetonitrile and then drying at room temperature.

### 3.2. The Autoxidation Process and Its Follow-Up by Weighing

The degree of oxidation of ML was monitored at room temperature in the light by weighing the sample on days 0 (=start of the experiment), 1–15, 19–23, 26–30, 33–36, 40, 42–43 and 47. The measurements were carried out during the light (summer) season between 9 June and 26 July 2010, in Helsinki, Finland. An amount of the ester was weighed into an open pre-weighed Petri dish made of laboratory glass (diameter *ca.* 5 cm) and the oxidation tests were started. An analytical balance, the precision of which was 0.01 mg, was used for weighing. The weight of the ML at the beginning of the experiment (after taking the initial 0-day sample for microbiological studies) was 1.88050 g. The sample was put in front of a window on a window board inside our laboratory in such a place where the sample was exposed to sunlight from two corner windows, one of which opened to the north-northwest and the other one to the east-northeast. The dish was kept under a protecting cover that formed a “roof” at a distance of *ca*. 10 cm above the dish and whose task was to protect the sample from dust, yet preventing neither exposure to daylight nor to air.

### 3.3. Microbial Strains. Determination of Antimicrobial Activity as a Function of the Autoxidation Process

Four different microbial strains (the yeasts *C. albicans* and *S. cerevisiae*, the Gram-negative bacterium *E. coli* and the Gram-positive bacterium *S. aureus*) were employed. These strains, their origin and the culture conditions and media used as well as the controls employed have been previously described in detail [8,9,10].

Inhibitory zone measurements were carried out using agar diffusion methodology according to “Method II” in [10]. Minimal inhibitory concentration (MIC) values were measured as previously described [11].

Samples for antimicrobial activity measurements (*ca.* 60–70 mg each) were taken from the material on the Petri dish on days 0–2, 5, 7, 9, 12, 20 and 34, and the Petri dish was weighed both before and after the sampling.

The samples were dissolved in dimethyl sulfoxide (DMSO). In the agar diffusion tests, the concentration of the test substance in DMSO was 40 mg/mL and 10 µL of this solution were pipetted onto each filter paper disc (diameter 6 mm). Thus, an “inhibitory zone” of 6 mm diameter indicates inactivity of the test substance.

In the MIC tests, the concentrations of the test substance in the medium were 1000, 500, 250, 125, 63, 31, 16, 7.8, 3.9, 2.0, 0.49 and 0.12 µg/mL. For yeasts, the test was continued for two days, the wells being observed also at 24 h. For bacteria, generation times are so much shorter than for yeasts that it is sufficient to use one-day cultures.

While studying the microtitration plates that contained the samples taken on days 0 and 1, an unexpected problem was encountered: those wells that contained the highest concentrations of the test substance (≥250 or, in some cases, 125 µg/mL) were covered by a lipid film that made it impossible to reliably determine growth by inspecting the plates in the normal manner previously described, and thus the results remained inconclusive for those concentrations.

We therefore tried to find a method for studying the presence or absence of microbial growth also in those wells that were covered with a marked lipid film. We found that in spite of the fact that the plates are made of a “non-transparent” plastic that prevents e.g., the use of plate readers, it is possible to verify or exclude the formation of a microbial pellet on the bottom of individual wells by observing the plates from beneath. Thus, for samples taken on day 5 or later, the microtitration plates were observed also from beneath in order to verify or exclude the formation of a microbial pellet on the bottom of individual wells (the formation of the pellet is a sign of growth, while the lack of it indicates growth inhibition). Thus, for samples taken on day 5 or later, results were obtained for all concentrations tested. A deep-frozen sample of the same lot of ML as was used for the present experiments (*i.e*., that is equivalent with the day 0 sample) was later studied so that the wells were observed also from beneath, and no growth inhibition was displayed.

We performed an extensive literature search but found no reports on studies of the antimicrobial effects of autoxidation products of polyunsaturated fatty acids. As concerns compounds formed by autoxidation of α-linolenic acid and other polyunsaturated fatty acids in plants and animals, however, numerous studies have been performed. Thus, Imbusch and Mueller [12] have shown that a series of dinor isoprostanes F1, termed phytoprostanes F1, are formed by non-enzymatic oxidation of alpha-linolenate in plants. Because the structurally related prostaglandins F2 and isoprostanes F2 exert potent biological activities, phytoprostanes might be involved in the antimicrobial activity that we found to occur in the early oxidative phase of the autoxidation process of ML. As discussed by Durand* et al.* [13], cyclopentenone phytoprostanes may be archetypal mediators of oxidative stress. Furthermore, very interestingly, Mueller’s group has shown [14] that in *Arabidopsis thaliana*, upon infection with the bacterium *Pseudomonas syringae*, an early accumulation of non-enzymatically synthesized hydroxy fatty acids and phytoprostanes F1 and, especially, of their esters occurs as part of the defence mechanisms of the plant against the microbe. Liu* et al*. [15] in turn have studied in detail several biologically active dihydroxylated fatty acid isomers formed from α-linolenic acid.

## 4. Conclusions

When ML was exposed to sunlight and air, its antimicrobial activity against yeasts and bacteria, as measured by agar diffusion, reached its maximum during the early oxidative phase when addition of oxygen occurred and the mass increased drastically. The activity increased rapidly during the first days of the exposure test, simultaneously with the increase of the mass of the material and began to decrease while the mass was still increasing due to the ongoing addition of oxygen and before the mass began to decrease due to degradation and formation of volatile compounds. Thus, the products formed at the late (degradation) phases of the process are far less toxic to the microbial strains studied than are the compounds formed at the early stages when addition of oxygen occurs with maximal rate.

## References

[1] Esterbauer H. (1993). Cytotoxicity and genotoxicity of lipid-oxidation products. Am. J. Clin. Nutr..

[2] Kubow S. (1990). Toxicity of dietary lipid peroxidation products. Trends Food Sci. Technol..

[3] Kubow S. (1992). Routes of formation and toxic consequences of lipid oxidation products in foods. Free Rad. Biol. Med..

[4] Kabara J.J., Swieczkowski D.M., Conley A.J., Truant J.P. (1972). Fatty acids and derivatives as antimicrobial agents. Antimicrob. Agents Chemother..

[5] Desbois A.P., Smith V.J. (2010). Antibacterial free fatty acids: Activities, mechanisms of action and biotechnological potential. Appl. Microbiol. Biotechnol..

[6] Matikainen J., Laantera M., Kaltia S. (2003). Determination of degree of oxidation of methyl linoleate and linolenate by weighing method. J. Am. Oil Chem. Soc..

[7] Kaltia S., Matikainen J., Ala-Peijari M., Hase T. (2008). Synthesis of ethyl 5*Z*,9*Z*,12*Z*-octadecatrienoate (ethyl pinolenate) and methyl 12*Z*,15*Z*-octadecadienoate. J. Am. Oil. Chem. Soc..

[8] Pelttari E., Matikainen J., Elo H. (2002). Antimicrobial activity of the marine alkaloids haminol and pulo’upone and related compounds. Z. Naturforsch. C.

[9] Pelttari E., Lehtinen M., Elo H. (2011). Substituted salicylaldehydes as potential antimicrobial drugs: Minimal inhibitory and microbicidal concentrations. Z. Naturforsch. C.

[10] Elo H. (2007). Antimicrobial activity of two antitumour agents and ribonucleotide reductase inhibitors, pyridine-2-carboxaldehyde thiosemicarbazone and the acetate form of its copper(II) chelate. Z. Naturforsch. C.

[11] Lehtinen M., Pelttari E., Elo H. (2011). Antimicrobial activity of formylchromones: Detection by a micro-scale method. Z. Naturforsch. C.

[12] Imbusch R., Mueller M.J. (2000). Formation of isoprostane F_2_-like compounds (phytoprostanes F_1_) from α-linolenic acid in plants. Free Radic. Biol. Med..

[13] Durand T., Bultel-Poncé V., Guy A., Berger S., Mueller M.J., Galano J.M. (2009). New bioactive oxylipins formed by non-enzymatic free-radical-catalyzed pathways: The phytoprostanes. Lipids.

[14] Grun C., Berger S., Matthes D., Mueller M.J. (2007). Early accumulation of non-enzymatically synthesised oxylipins in *Arabidopsis thaliana* after infection with *Pseudomonas syringae*. Funct. Plant Biol..

[15] Liu M., Chen P., Véricel E., Lelli M., Béguin L., Lagarde M., Guichardant M. (2013). Characterization and biological effects of di-hydroxylated compounds deriving from the lipoxygenation of ALA. J. Lipid Res..

